# Estrogen Receptor Mutations as Novel Targets for Immunotherapy in Metastatic Estrogen Receptor–positive Breast Cancer

**DOI:** 10.1158/2767-9764.CRC-23-0244

**Published:** 2024-02-22

**Authors:** Jonathan Goldberg, Na Qiao, Jennifer L. Guerriero, Brett Gross, Yagiz Meneksedag, Yoshimi F. Lu, Anne V. Philips, Tasnim Rahman, Funda Meric-Bernstam, Jason Roszik, Ken Chen, Rinath Jeselsohn, Sara M. Tolaney, George E. Peoples, Gheath Alatrash, Elizabeth A. Mittendorf

**Affiliations:** 1 Division of Breast Surgery, Department of Surgery, Brigham and Women's Hospital, Boston, Massachusetts.; 2 Breast Oncology Program, Dana-Farber Brigham Cancer Center, Boston, Massachusetts.; 3 Department of Hematopoietic Biology & Malignancy, University of Texas MD Anderson Cancer Center, Houston, Texas.; 4 Harvard Medical School, Boston, Massachusetts.; 5 Faculty of Medicine, Hacettepe University, Ankara, Turkey.; 6 McGovern Medical School, University of Texas Health Science Center at Houston, Houston, Texas.; 7 Department of Investigational Cancer Therapy, The University of Texas MD Anderson Cancer Center, Houston, Texas.; 8 Sheikh Khalifa Bin Zayed Al Nahyan Institute for Personalized Cancer Therapy, The University of Texas MD Anderson Cancer Center, Houston, Texas.; 9 Department of Genomic Medicine, the University of Texas MD Anderson Cancer Center, Houston, Texas.; 10 Department of Bioinformatics and Computational Biology, The University of Texas MD Anderson Cancer Center, Houston, Texas.; 11 Department of Medical Oncology, Dana-Farber Cancer Institute, Boston, Massachusetts.; 12 LumaBridge (formerly Cancer Insight), San Antonio, Texas.; 13 Department of Stem Cell Transplant and Cellular Therapy, University of Texas MD Anderson Cancer Center, Houston, Texas.

## Abstract

Estrogen receptor–positive (ER^+^) breast cancer is not considered immunogenic and, to date, has been proven resistant to immunotherapy. Endocrine therapy remains the cornerstone of treatment for ER^+^ breast cancers. However, constitutively activating mutations in the estrogen receptor alpha (*ESR1*) gene can emerge during treatment, rendering tumors resistant to endocrine therapy. Although these mutations represent a pathway of resistance, they also represent a potential source of neoepitopes that can be targeted by immunotherapy. In this study, we investigated *ESR1* mutations as novel targets for breast cancer immunotherapy. Using machine learning algorithms, we identified *ESR1*-derived peptides predicted to form stable complexes with HLA-A*0201. We then validated the binding affinity and stability of the top predicted peptides through *in vitro* binding and dissociation assays and showed that these peptides bind HLA-A*0201 with high affinity and stability. Using tetramer assays, we confirmed the presence and expansion potential of antigen-specific CTLs from healthy female donors. Finally, using *in vitro* cytotoxicity assays, we showed the lysis of peptide-pulsed targets and breast cancer cells expressing common *ESR1* mutations by expanded antigen-specific CTLs. Ultimately, we identified five peptides derived from the three most common *ESR1* mutations (D538G, Y537S, and E380Q) and their associated wild-type peptides, which were the most immunogenic. Overall, these data confirm the immunogenicity of epitopes derived from *ESR1* and highlight the potential of these peptides to be targeted by novel immunotherapy strategies.

**Significance:**

Estrogen receptor (ESR1) mutations have emerged as a key factor in endocrine therapy resistance. We identified and validated five novel, immunogenic ESR1-derived peptides that could be targeted through vaccine-based immunotherapy.

## Introduction

Approximately 70% of breast cancers express the estrogen receptor (ER), rendering them susceptible to endocrine therapy (1, 2). Despite the initial response to endocrine therapy, many patients with metastatic disease eventually progress on endocrine therapy (3). Endocrine resistance evolves through many mechanisms including genetic dysregulation, posttranslational modifications, altered cell signaling, ligand-independent activation of the ER, and decreased sensitivity to antiestrogens. A major mechanism of resistance is mutation of the ERα gene *ESR1*, which occurs in approximately 30% of patients after treatment with aromatase inhibitors (AI; refs. 3–7). Several *ESR1* mutations have been identified, the majority of which occur in the ligand-binding domain (6). The most common mutations, D538G, Y537S, and E380Q, are associated with poor therapeutic response and overall survival (3, 5, 8). In addition, preclinical data show that these mutations are drivers of metastases, and thus targeting these mutations could lead to improved outcomes (9). In this study, we aimed to generate and validate an immune-based approach for targeting these mutations that could lead to improved outcomes and restore endocrine sensitivity.

There is growing interest in using tumor-expressed mutations to inform immunotherapeutic approaches. The total number of mutations present within a tumor [i.e., tumor mutational burden (TMB)] is an emerging biomarker that has been shown to independently predict the response to immunotherapy in multiple cancer types (10–12). However, the TMB of breast cancer is relatively low, especially in ER-positive (ER^+^) breast cancer (13, 14). However, specific mutations can lead to the generation of neoantigens that are expressed by malignant cells and are critical for distinguishing tumors from normal cells. Tumor-expressed mutations have been exploited as potential targets for T cell–based and vaccine-based approaches. In addition to mutated epitopes, several studies (15, 16), including our work (17–20), have shown that non-mutated self-antigens are effective therapeutic targets. Importantly, *ESR1* expression is higher in breast cancer tissues than in other malignant and healthy tissues, including healthy breast tissues. Therefore, our interest in ER as a target for immunotherapy stems from the fact that wild-type *ESR1* itself can be a source of self-antigens and *ESR1* can be mutated, making it a potential source of neoantigens.

Using publicly available machine learning algorithms, we identified novel peptides derived from *ESR1* that can present to CD8^+^ T cells. To validate these candidate peptides, we used *in vitro* T2 binding assays to measure peptide affinity for HLA-A*0201, and dissociation assays to measure the stability of these complexes on the cell surface. Next, we focused on the ability of high-affinity peptides to induce an endogenous cytolytic CD8^+^ T-cell response in healthy volunteers. Overall, we identified five novel peptides derived from wild-type and mutant *ESR1* with a high affinity for HLA-A*0201 that elicited a strong CD8^+^ T-cell response. Together, our data support the potential of *ESR1* candidate peptides as T-cell targets in vaccine or adoptive T-cell therapy approaches.

## Materials and Methods

### Peptide Preparation

*ESR1* gene sequencing was performed using the NIH gene database NM_000125.4, and clinically relevant *ESR1* mutations were confirmed from previous studies. To identify potential HLA-A*0201 binding peptides within *ESR1* mutation sites, the Immune Epitope Database and Analysis Resource (IEDB) at iedb.org was used (21), which has been used in our previous work and in similar investigations into ESR1 peptides (22). Candidate peptides with low IC_50_ scores (<500 nmol/L) predicted by ANN, SMM, and NetMHCpan algorithms, along with those peptides validated through the overlapping peptide approach, were synthesized at Bio-Synthesis and processed to at least 95% purity by reverse-phase high-performance liquid chromatography and confirmed by mass spectrometry. Influenza peptide (FLU: GILGFVFTL) was used as a positive control, and each mutant peptide's corresponding wild-type sequence was used as a negative control. Lyophilized peptides were dissolved in PBS containing 5% DMSO at a concentration of 1 mg/mL and stored at −80°C.

### Cell Lines and Flow Cytometry Analysis

The T2 and MCF7 cell lines were purchased from the ATCC. Prior to use, these cell lines were validated using short tandem repeat DNA fingerprinting at the MD Anderson Cancer Center sequencing facility. MCF7-mutant cell lines were gifted from Dr. Rinath Jeselsohn. After thawing, cells were used for experiments within 5 passages. *Mycoplasma* testing was performed quarterly using the MycoAlert Kit (Lonza Inc.). All cell lines were maintained in RPMI1640 media supplemented with 10% heat-inactivated FBS (Gemini BioProducts), 100 U/mL penicillin, and 100 µg/mL streptomycin (Gibco-Invitrogen). All cells were cultured at 37°C and 5% CO_2_. Cells were pulsed with or without peptides at designated timepoints, harvested, and stained with a FITC-conjugated anti-HLA-A2 mAb (BB7.2, BD Biosciences). Live/dead Aqua staining (Life Technologies) was used to assess the cell viability. Flow cytometry was performed using a BD LSRFortessa flow cytometer (BD Biosciences), and the data were analyzed using FlowJo software (TreeStar Inc.).

### Peptide Binding Assay and Stability Assay

T2 binding assays were performed as described previously (23). Briefly, to test peptide binding affinity to HLA-A*0201 molecules, T2 cells were incubated with 40 µg/mL of mutant, wild-type, or control peptides for 4 hours at 37°C in RPMI1640 media supplemented with 0.5% FBS. Surface expression of HLA-A*0201 in T2 cells was determined by staining with an anti-HLA-A2 mAb (BB7.2, BD Biosciences). The median fluorescence intensity (MFI) was measured using a FACSCando II (BD Biosciences). The peptide half-life was calculated by linear regression using GraphPad Prism software.

### Peptide-specific CTL Expansion and Tetramer Assay

Healthy donor peripheral blood samples were purchased from the local blood bank and used under an MD Anderson Cancer Center Institutional Review Board–approved protocol. Because the samples came from deidentified healthy donors, the Institutional Review Board determined that informed consent was not required. Peripheral blood mononuclear cells (PBMC) were isolated from buffy coats of healthy female HLA-A2 positive donors by Ficoll/Histopaque (Sigma-Aldrich) density gradient centrifugation and split equally into two groups. One group of PBMC was used to assess the pre-expansion frequency of the peptide-specific CTLs. These cells were immediately stained with FITC-conjugated anti-CD3 and APC-H7–conjugated anti-CD8 to select for CD8^+^ T cells. Pacific blue (PB)-conjugated anti-CD4, anti-CD14, anti-CD16, anti-CD19, and anti-CD56 (BD Biosciences) were used as a dump gate to gate out other immune cell types; PE-conjugated mutant peptide and APC-conjugated wild-type peptide tetramers (Baylor College of Medicine, MHC tetramer core, Houston, TX) were used to enumerate the percentage of peptide-specific CD8^+^ T cells present in healthy PBMCs. The analysis was performed using flow cytometry.

The second group of donor PBMC was used to assess the expansion potential of the peptide-specific CTLs. To expand peptide-specific CTLs, dendritic cells (DC) were matured from adherent monocytes by the addition of GMCSF (100 ng/mL, Sanoti), IL4 (50 ng/mL, rhIL4, Tonbo Biosciences), and TNFα (25 ng/mL, BioLegend). Lymphocytes from the same donor were separated and cocultured with 40 µg/mL of mutant and wild-type peptides. After 5 days, DCs were harvested and cocultured with autologous CD8^+^ T cells for an additional 7 days. The cocultured cells were supplemented with IL7 (10 ng/mL; BioLegend) for CTL activation and IL2 (25 ng/mL; R&D Systems) for CTL expansion. After CTL activation and expansion, cells were stained and analyzed as described above.

### Peptide-specific CTLs Cytotoxicity Assay

Healthy female donor lymphocytes and DCs were cocultured in 6-well plates supplemented with IL7 and IL2, as described above. On days 12–14, cultured peptide-specific CTLs were harvested and peptide-specific cytotoxicity was assessed using a standard 4-hour Calcein-AM (Sigma-Aldrich) release assay as described previously (24). Briefly, target cells, including T2 cells pulsed with mutant and wild-type peptides, were labeled with 5 µg/mL calcein-AM for 15 minutes in the dark. After two washes, cells were adjusted to 2 × 10^5^/mL, plated on a Terasaki plate, and cocultured with peptide-specific CTLs at effector:target ratios of 20:1 and 5:1. After 4 hours of incubation, Trypan blue was added to quench the reaction. The fluorescence was measured using a Cytation 3 Imaging Reader (BioTek). The percentage of specific cell lysis was calculated using the following formula:



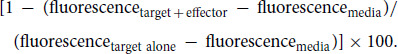



### Statistical Analysis

Statistical analyses were performed using the GraphPad Prism, version 8.0. Data were compared using paired Student *t* test. A probability of less than 0.05 was considered significant at *, *P* < 0.05; **, *P* < 0.01; ***, *P* < 0.001.

### Ethical Compliance

All procedures performed in this study involving human participants were in accordance with the ethical standards of the Institutional and/or National Research Committee and with the 1964 Helsinki Declaration and its later amendments or comparable ethical standards.

### Data Availability

The data generated in this study are available upon request from the corresponding author.

## Results

### Multiple HLA-A*0201 Binding Epitopes are Identified

We hypothesized that ESR1 mutations would generate novel peptides with high binding affinities for HLA class I molecules. To determine which epitopes derived from within ESR1 mutation sites would have a high propensity to bind to HLA-A*0201 (IC_50_ < 500 nmol/L; ref. 25), IEDB-binding algorithms were used to identify 10 nonameric/decameric peptide sequences containing ESR1 mutations, as well as their associated wild-type peptides (Supplementary Table S1). These peptides had a predicted IC_50_ of <500 nmol/L using at least two separate algorithms. We validated multiple ESR1 peptides *in vitro*, including a peptide derived from the E380Q mutation. However, IEDB-predicted peptides for the two most common mutations, D538G and Y537S, failed downstream biological validation. Given the frequency of D538G and Y537S mutations and their potential clinical value as immunotherapy targets, we next pursued an overlapping peptide approach in which we tested every octameric, nonameric, and decameric permutation of peptides that included D538G and Y537S point mutations. Although IC_50_< 500 nmol/L is the conventional cutoff for predicting avid peptide binding to HLA-A201 and effective T-cell recognition (26), a number of peptides, including MART1, gp100, and GP2, have been shown to be effective immunotherapy targets despite a predicted IC_50_ > 500 nmol/L (refs. 27–29; Supplementary Table S2). Ultimately, in our analysis, nonameric peptides were predicted to be better binders than octomeric or decameric peptides for both Y537S and D538G. Thus, we proceeded with nonameric overlapping peptides for both Y537S and D538G mutations (Supplementary Table S3). The top three peptides for the Y537S and D538G mutations were selected on the basis of the predicted IC_50_ (lowest IC_50_ from an individual algorithm) and subsequently validated using *in vitro* T2 binding assays (Supplementary Fig. S1). By analyzing the overlapping peptides, we established a high-affinity peptide for both D538G and Y537S.

Although our initial *in silico* approach yielded several high-affinity peptides derived from multiple ESR1 mutations, we focused on targeting mutations that are consistently and frequently identified in clinical settings. Analysis of data derived from the MSK-IMPACT, PALOMA-3, SOFEA, and FERGI trials revealed that 60%–80% of ESR1 mutations involve E380Q, Y537S, and D538G (ref. 30; Supplementary Table S4). In addition to the mutated peptides, we also included their respective wild-type peptides to serve both as a negative control and a potential immunotherapeutic target, as ESR1 is a highly enriched tumor-associated antigen (TAA) in ER^+^ breast tumors (Supplementary Fig. S2). Because Y537S and D538G are derived from the same wild-type sequence, only one wild-type control was required. Therefore, we ultimately established five novel ESR1-derived peptides for downstream validation: three from the most common ESR1 mutations and two from their associated wild-type sequences (Table 1).

**TABLE 1:** Identification of candidate ESR1-derived peptides

		IC_50_ (nmol/L)		
Peptide	Sequence	NetMHCpan 4.0	ANN	SMM
Wildtype-1	LLECAWLEI	379.1	394.08	432.62
E380Q	LLQCAWLEI	100.7	99.42	142.6
Wildtype-2	PLYDLLLEM	170.3	353.62	255.33
Y537S	PLSDLLLEM	907	1533.32	575.59
D538G	PLYGLLLEM	347.2	1059.68	554.77

### Candidate Peptides Bind to HLA-A*0201 with High Affinity and Stability

**FIGURE 1:**
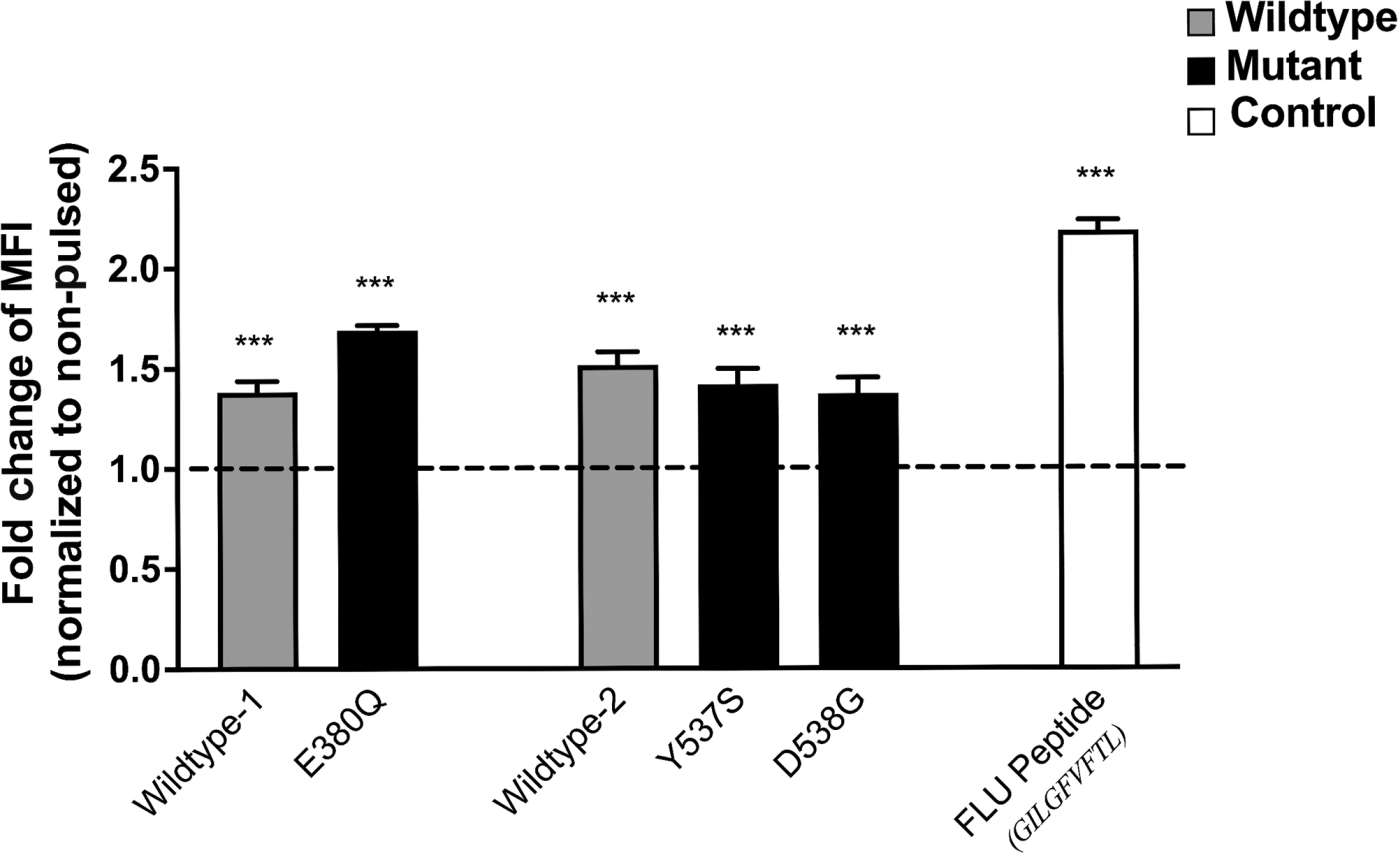
Peptides bind to HLA-A*0201. T2 cells were pulsed with indicated peptide for 4 hours and surface HLA-A*0201 was stained. MFI of pulsed T2 cells were normalized to experimentally matched non-pulsed T2 cells, and data expressed as fold change in MFI*.* Dashed horizontal line indicates non-pulsed T2 HLA-A*0201. FLU peptide was used as a positive control HLA-A*0201 peptide known to bind with high affinity. Data represent three independent experiments performed in triplicate. Statistical significance was determined via comparison of pulsed MFI versus non-pulsed raw MFI using unpaired Student *t* test. ***, *P* < 0.001.

**FIGURE 2:**
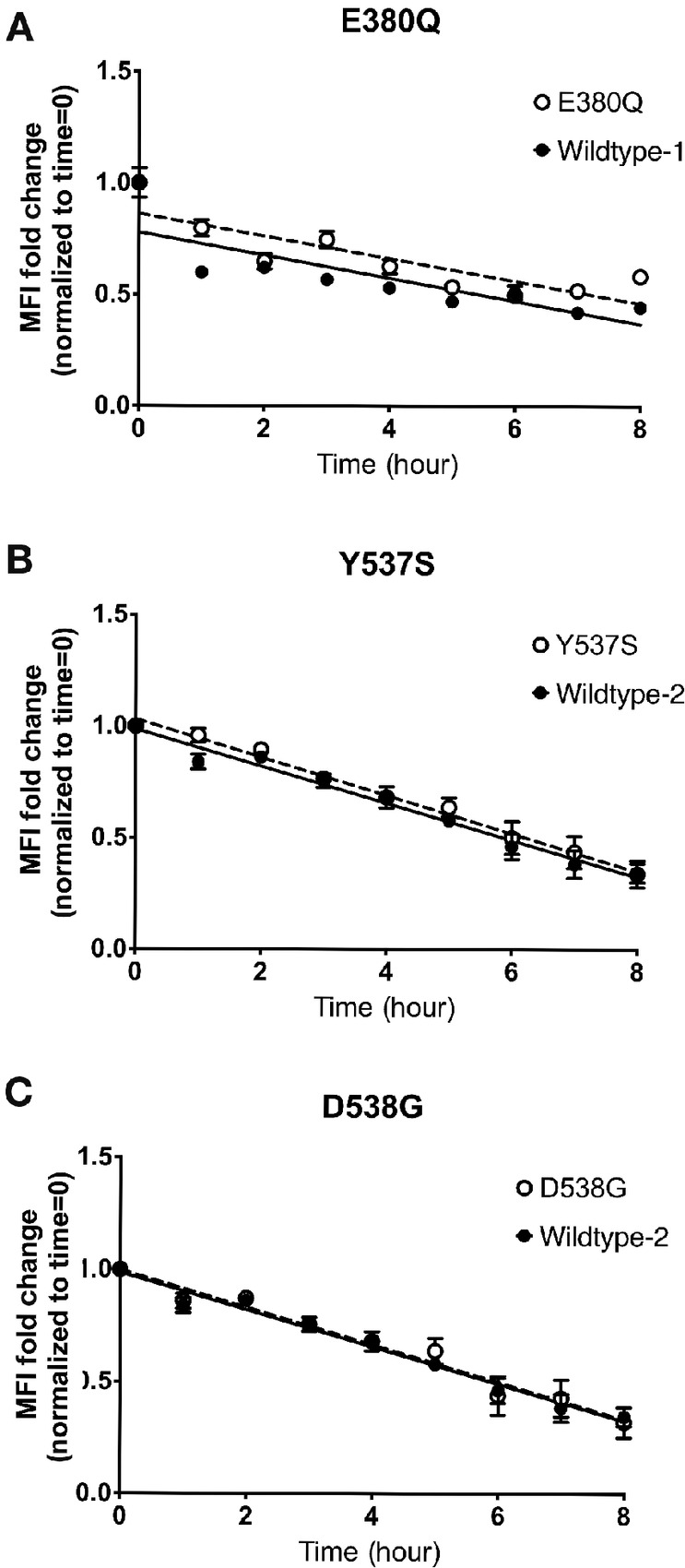
Kinetics of peptide: HLA-A*0201 dissociation. T2 cells were pulsed with indicated peptides for 8–12 hours, washed, and then stained for surface HLA-A*0201 expression at indicated timepoints for wildtype-1 and E380Q (**A**), wildtype-2 and Y537S (**B**), wildtype-2 and D538G (**C**). MFI for each timepoint was normalized to initial staining (time = 0). Linear regression analysis was used to plot lines of best fit for each individual peptide, and half-life for each peptide is shown in Table 2. Data are pooled from three independent experiments performed in triplicate.

To verify the HLA-A*0201 binding affinity of the five candidate peptides, T2 binding assays were performed. In comparison with non-pulsed T2 cell controls, the five peptides showed an average fold change in MFI of 1.5 (range: 1.38–1.69), indicating high-affinity peptide binding to surface HLA-A*0201 on T2 cells (Fig. 1). Importantly, previous studies have identified peptide affinity measured using biological assays as the strongest predictor of a robust CD8^+^ T-cell response (31, 32). T2 dissociation assays were used to determine the half-life and dissociation rates of peptides from HLA-A*0201 molecules. The results showed that these five peptides had half-lives ranging from 5.4 to 7.2 hours (Fig. 2; Table 2). There was a trend toward a shorter half-life in the wild-type peptides compared with the mutant peptides; however, all peptides showed a half-life of over 5 hours, which has been associated with an increased likelihood of immunogenicity (33–35). Overall, these data provide biological evidence that the predicted peptides can bind HLA-A*0201 with both high affinity and prolonged dissociation kinetics.

**TABLE 2:** Stability of candidate peptides to HLA-A*0201

Peptide	Half-Life (hours)
Wildtype-1	5.4
E380Q	7.2
Wildtype-2	5.9
Y537S	6.2
D538G	6.0

### ESR1 Peptide CTLs Can be Expanded from Healthy Female Donors

**FIGURE 3:**
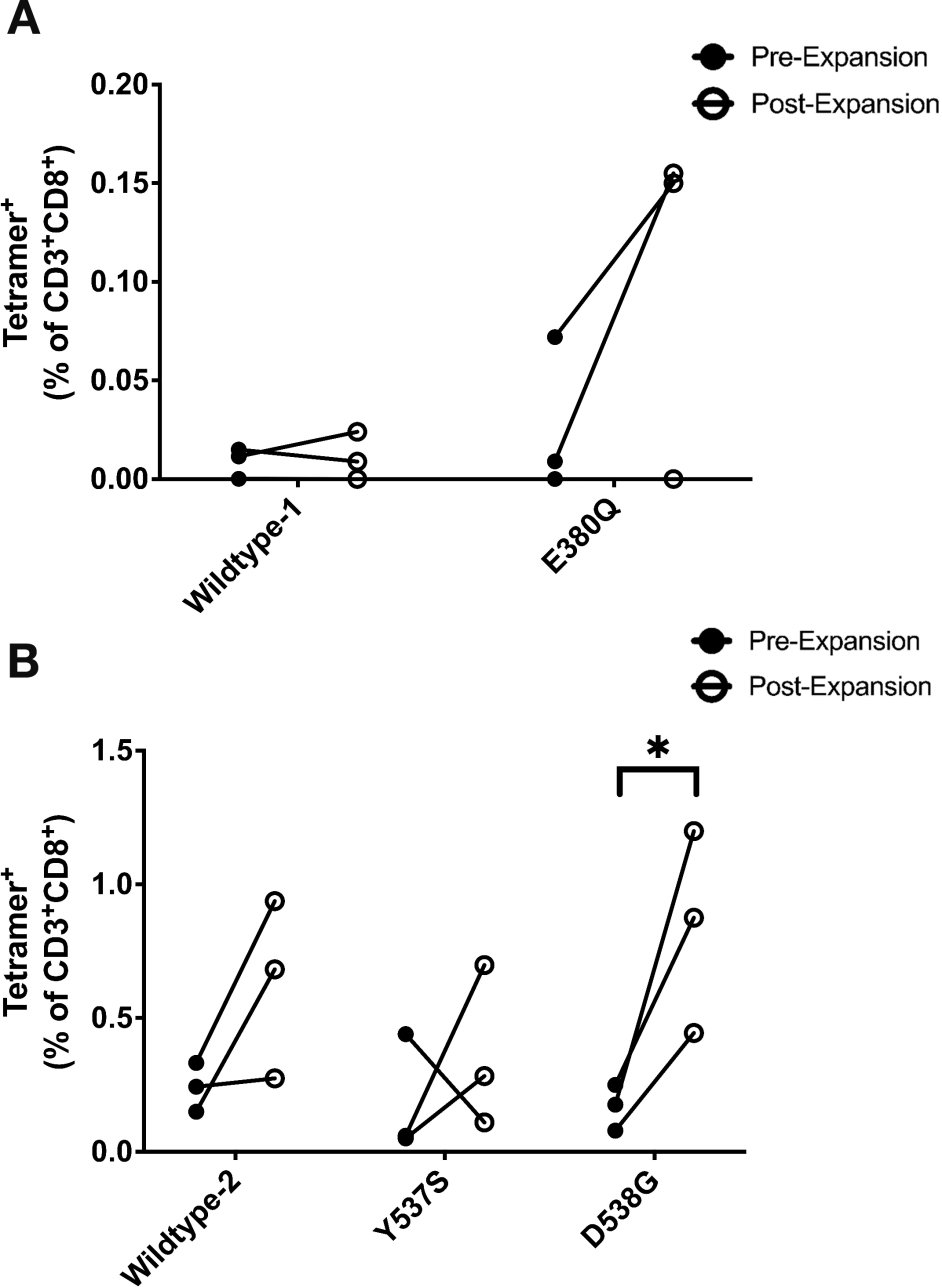
Peptide-specific expansion of CD8^+^ T cells. PBMCs from healthy donors were isolated and expanded as described in the Materials and Methods. Tetramer staining was performed pre- and post-expansion to quantify frequencies of peptide-specific CD8^+^ T cells. **A,** Wildtype-1, E380Q. **B,** Wildtype-2, Y537S, D538G. Data represents three independent experiments, from 3 separate donors, performed in triplicate. Statistical significance was determined via comparison of pre-expansion versus post-expansion CD8^+^tetramer^+^ population using unpaired Student *t* test. *, *P* < 0.05.

Having shown that these peptides can be presented for immune recognition complexed with HLA-A*0201, we next tested whether a CD8^+^ T-cell response that recognizes these specific peptide–HLA complexes can be detected in the peripheral blood. To confirm the immune recognition ability of the five candidate peptides, peptide-specific tetramer staining was performed on peripheral blood samples from multiple healthy female donors. These data revealed an average frequency of E380Q (0.03%), Y537S (0.18%), and D538G (0.17%)-specific CTLs in the peripheral blood of healthy females (Fig. 3), which is comparable to the precursor frequency of well-established, clinically relevant peptide tumor targets (36). Next, we aimed to determine whether peptide-specific CTLs can be expanded from healthy female donors. CTL expansion and subsequent tetramer staining revealed that mutant peptide-specific CTLs could be expanded, as shown by the average frequency fold changes (vs. pre-expansion) for E380Q (5.77-fold), Y537S (4.85-fold), and D538G (4.31-fold). Wildtype-1 did not show tetramer expansion, while wildtype-2 showed modest expansion compared with that of the mutant peptides. Overall, 4/5 candidate peptides yielded tetramer expansion, implying the potential of these peptides to elicit CD8^+^ T-cell immunity.

### ESR1 Peptide-specific CTLs Lyse ESR1 Peptide Pulsed T2 Cells

**FIGURE 4:**
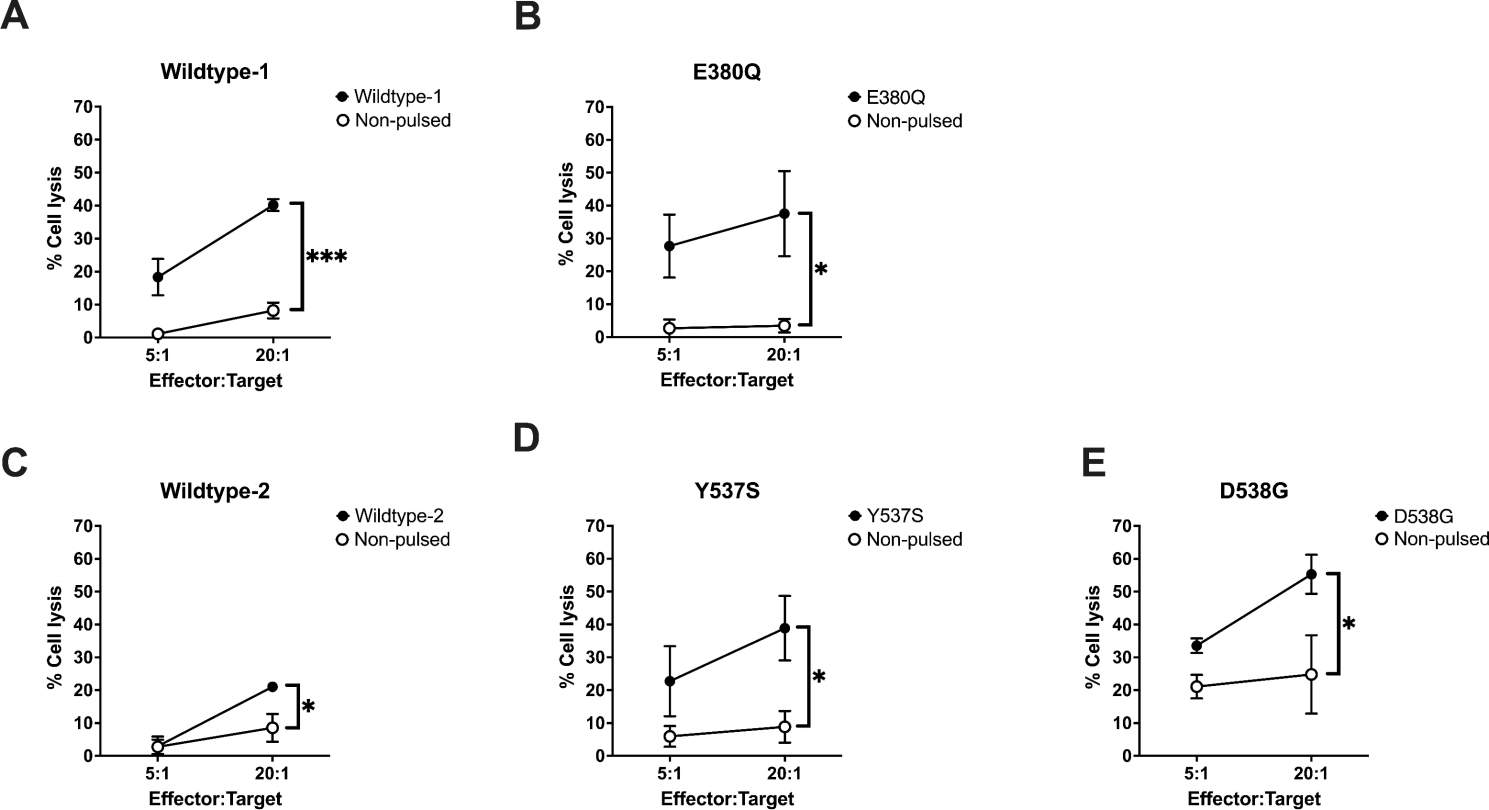
Expanded CD8^+^ T cells demonstrate antigen-specific cytotoxicity. T2 cells were pulsed with corresponding peptides and labeled with calcein-AM. Cytotoxicity was determined by a standard calcein-AM release assay. Wildtype-1 (**A**), E380Q (**B**), Wildtype-2 (**C**), Y537S (**D**), D538G (**E**). Non-pulsed T2 cells were used as negative controls. Statistical significance was determined via unpaired Student *t* test. Data represent the average of three to four experiments from separate healthy female donors run in triplicate. *, *P* < 0.05; ***, *P* < 0.001.

After confirming high-affinity binding, long half-lives, and peptide-specific CTLs in peripheral blood, we next aimed to determine whether targets expressing the candidate peptides could be lysed by peptide-specific CTLs. Peptide-specific CTLs were isolated and expanded from healthy female donors. T2 cells were pulsed with peptides as target cells, and peptide-specific CTL-mediated lysis was assessed using a calcein-AM cytotoxicity assay. The data revealed that all peptide-specific CTLs from both mutant and wild-type peptides lysed the T2 target cells pulsed with the corresponding peptide at an increasing effector:target ratio (Fig. 4). At 20:1 effector:target ratio, the E380Q, Y537S, and D538G peptides generated specific cytotoxicities of 28.45%, 30.03%, and 30.48%, respectively. Wildtype 1 and wildtype 2 also showed specific cytotoxicity of 25.99% and 13.27%, respectively. Taken together, these data confirm peptide-specific lysis, further validating the immunogenic potential of these five novel peptides.

### Peptide-specific CTLs Effectively Kill ER^+^ Breast Tumor Cells

**FIGURE 5:**
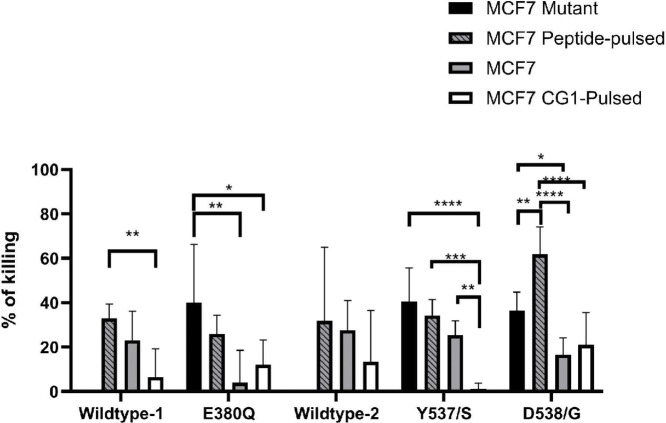
Peptide-specific CTLs effectively kill ER^+^ breast tumor cells. MCF7 cells were pulsed with corresponding peptides and labeled with calcein-AM. Cytotoxicity was determined by a standard calcein-AM release assay. MCF7 cells were pulsed with corresponding peptides. CG1-pulsed MCF7 cells and non-pulsed MCF7 cells were used as negative controls. Statistical significance was determined via one-way ANOVA. Data show percent cytotoxicity at the 10:1 effector:target ratio, and represent the average cytotoxicity of five experiments (run in triplicate) from different healthy female donors. *, *P* < 0.05; ***, *P* < 0.001.

We next aimed to identify whether breast cancer cells show similar cytotoxicity to peptide-specific CTLs as we demonstrated for peptide-pulsed T2 cells. Peptide-specific CTLs were isolated and expanded from healthy female donors. MCF7 wild-type cells were pulsed with peptides and served as the target cells. Peptide-specific CTL-mediated lysis was assessed using a calcein-AM cytotoxicity assay. Non-pulsed and CG1-peptide pulsed MCF7 cells were used as negative controls. In addition, mutant MCF7 cells for each of the three mutants, E380Q, Y537S, and D538G, were also evaluated for killing by corresponding mutant peptide-specific CTL (Fig. 5). These data showed increased killing of peptide-pulsed MCF7 cell lines by the appropriate peptide-specific CTLs compared with controls. In addition, our data demonstrate cytotoxicity of mutant ESR1 cell lines, comparable to what was observed with peptide-pulsed wild-type MCF7 cell lines.

## Discussion

In this study, we provided evidence that T-cell immunity can be generated against ESR1-derived peptides. We identified multiple candidate peptides using well-defined machine learning algorithms, and subsequently identified and systematically validated five ESR1-derived nonameric peptides that bind with high affinity and form a stable complex with HLA-A*0201. We then detected ESR1 peptide-specific CTLs in the peripheral blood of healthy females and demonstrated their expansion. Finally, using functional assays, we demonstrated that peptide-specific CTLs lysed peptide-pulsed T2 targets as well as MCF7 cell lines. Taken together, these data support further investigation of immunotherapeutic strategies, including vaccines or adoptive T-cell therapy approaches targeting ESR1.

Advances in early screening and extended regimens of endocrine therapy have partly led to improved outcomes in patients with ER^+^ breast cancer. For postmenopausal patients, therapy with AIs for 5–10 years is standard (37). AIs, such as anastrozole, prevent the synthesis of estrogen and starve the ER of its ligand. Under the selective pressure of AIs, tumors are driven to develop endocrine resistance through multiple pathways, with ESR1 mutations being the most common genetically acquired pathway. In the metastatic setting, approximately 30% of patients who progress on AIs develop one or multiple ESR1 mutations (6). At least 62 *ESR1* mutations have been identified; however, the majority of these mutations are rarely identified and are either inactive or yet to be characterized (6). Thus, we focused our investigations on the three most common ESR1 mutations (E380Q, Y537S, and D538G), which are robustly characterized and directly linked to poor outcomes (3, 8, 38). By focusing on the most common mutations, we anticipate that further development of immunotherapeutic strategies targeting these mutations will yield the greatest benefits to patients.

The E380Q, Y537S, and D538G mutations represent the source of neoantigens in the limited mutational landscape of ER^+^ breast tumors. While neoantigen load is predictive of T-cell infiltration in multiple cancers, including breast cancer, Williams and colleagues found that ESR1 mutations did not increase endogenous CD8^+^ T-cell infiltration. Conversely, ER^+^ tumors with other common mutations, such as TP53 and PIK3CA, show significantly elevated levels of CD8^+^ T-cell infiltration (39). One hypothesis for this lack of T-cell response to ESR1 mutations is the poor binding of endogenously processed peptides to MHC class I. Our initial *in silico* approach failed to identify high-affinity peptides for the Y537S and D538G mutations, which may indicate that this region of the ligand-binding domain has certain qualities that limit antigen presentation. Strong binding to MHC molecules relies on peptide length and specific anchor residues. For HLA-A2, peptides comprised of nine amino acids with leucine at anchor residue 2 confer enhanced binding (40). Each ESR1-mutated peptide presented here meets both of these criteria, which may, in part, explain their high binding affinity to HLA-A*0201.

As part of our initial experimental design, we aimed to use wild-type peptides as negative controls, from which we compared the corresponding mutant peptides. Interestingly, the two wild-type “controls” displayed similar binding characteristics to that of the mutant peptides and were similarly able to elicit a strong and specific T-cell response. Although neoepitopes, such as ESR1 mutations, are known for their enhanced immunogenicity, we hypothesize that the peptide sequences discovered in this study may have inherent properties, rendering them sufficiently immunogenic, with or without mutations. Moreover, highly overexpressed wild-type proteins can also be TAAs that are recognized by the immune system and have been successfully targeted in multiple cancer types (15–18). In ER^+^ breast cancer, wild-type ERα is a highly enriched TAA. IHC studies have revealed significantly higher levels of ERα in malignant mammary epithelial cells than in benign mammary epithelial cells. Furthermore, Khan and colleagues found that ER positivity in benign mammary tissues was correlated with an increased risk of breast cancer (41, 42). On the basis of these data, in addition to mutated ESR1 peptides, we included their corresponding wild-type epitopes as potential immunotherapeutic targets. Importantly, these wild-type peptides had HLA-binding properties and immunogenic potential comparable to those of their respective mutant epitopes.

The selective ER degrader (SERD) fulvestrant, often used as a second-line therapy for metastatic ER^+^ breast cancer, has a weaker binding affinity to mutated ER compared with wild-type receptors (38). The poor bioavailability of intramuscularly administered fulvestrant further limits the effective targeting of mutant ER. Importantly, newer agents are in development including oral SERDs, which have better absorption and increased bioavailability, enabling enhanced antagonist activity (43). In addition, there are selective ER modulators and SERDs that have been shown to have a structure that destabilizes mutant ER. (44) Small-molecule therapeutics, such as mTOR and CDK4/6 inhibitors, have shown some efficacy against ESR1-mutated tumors (6, 37); however, these therapies, combined with standard therapy, can lead to significant toxicities and disease progression is nearly always inevitable. The phase III PADA-1 trial demonstrated that switching to fulvestrant as the partner endocrine treatment with the CDK 4/6 inhibitor palbociclib was superior to continuing on an AI with palbociclib in the setting of rising ctDNA ESR1 mutations (45). These data provided evidence that better blockade of ESR mutations during treatment with endocrine therapy plus a CDK 4/6 inhibitor improved outcomes. Moreover, once patients progress with endocrine therapy, they are likely to cycle through other treatments, ultimately progressing to chemotherapy.

Our findings pave the way for the development of ESR1 targeted immunotherapy. One approach could be vaccine development, as we were able to expand CD8^+^ T cells to target these five novel peptides. Importantly, vaccination against immunogenic epitopes has been shown to successfully generate a CD8^+^ T-cell response against another cancer relevant protein, HER2. E75, a nanomeric peptide derived from HER2, has been used in multiple clinical trials for breast, ovarian, and gastric cancers, many of which were led by our group. In preclinical development, E75 displayed high affinity and stability for HLA-A*0201 and could be recognized by and prime CTLs (46). In a phase I study, vaccination with E75 in 14 patients with metastatic HER2^+^ breast cancer resulted in immunologic responses in the majority of patients (47). In a subsequent phase I/II trial enrolling 187 patients with high-risk HER2^+^ breast cancer, E75 vaccination in an adjuvant setting reduced the relative risk of recurrence by 48% (48). A subsequent multicenter phase III trial (NCT 01479244) targeting patients with HER2-low–expressing cancer (1–2+ by IHC) failed to demonstrate the clinical benefit of E75 vaccination as monotherapy (49). To explain the negative phase III trial data with the E75 vaccination, we hypothesized that a vaccine comprised of a single-epitope vaccine is insufficient to consistently generate an antitumor response. Therefore, in our current effort we are pursuing a multiepitope vaccine strategy that incorporates the five immunogenic epitopes identified in this study. The goal of such a vaccine strategy would be to educate CD8^+^ T cells to identify and eliminate circulating tumor cells that may contain *ESR1* mutations which could potentially extend the length of time patients respond to endocrine therapy. Importantly, other groups are now investigating peptide vaccines targeting ESR1, including a phase I trial evaluating a vaccine comprised of peptides and an immunoadjuvant (either montanide or GMCSF; NCT04270149).

A second therapeutic approach would be cell-based treatment that utilizes T-cell receptor (TCR)-engineered T cells to target ESR1 peptides, as has been done for gp100 and MART-1 peptides in the setting of metastatic melanoma (50) and for MAGE-A4 peptide in the setting of esophageal cancer (7). With the knowledge of peptide–HLA complexes, a third approach could utilize a TCR mimic (TCRm) antibody to target ESR1 peptide–HLA complexes, as was shown preclinically in breast cancer models with E75 peptide targeting TCRm antibody (19) and in the setting of leukemia targeting WT1 and PR1 peptides with TCRm antibodies (51, 52).

In conclusion, we show the presence of immunogenic epitopes within the most common mutations of ESR1 as well as the wild-type ESR1 protein. CTL specific for these epitopes can recognize and lyse target cells expressing the mutations. A therapeutic strategy targeting these mutations with immunotherapy such as a multiepitope vaccine warrants further preclinical and clinical investigation.

## Supplementary Material

Supplementary Figure S1 Kinetics of Overlapping peptide

Supplementary Table S1 Initial in-silico identification of 18 novel peptides with predicted high affinity to HLA-A*0201 (IC50<500 nM)

Supplementary Figure S2 Expression of ESR1 in various Healthy and Malignant Tissues

Supplementary Table S2 Clinically Validated Peptides with IC50>500nM

Supplementary Table S3 Overlapping nonameric peptides in-silico prediction

Supplementary Table S4 Frequency of most common ESR1 mutations in key clinical studies
