# Supplementary material for: Estrogen Receptor Mutations as Novel Targets for Immunotherapy in Metastatic Estrogen Receptor–positive Breast Cancer
Source: Cancer Res Commun. 2024 Feb 22;4(2):496–504. doi: 10.1158/2767-9764.CRC-23-0244 (PMC10883292; doi:10.1158/2767-9764.CRC-23-0244)

Supplementary Figure S1

Kinetics of Overlapping peptide: HLA-A*0201 dissociation Nonameric overlapping peptides for both Y537S and D538G mutations with high predictive binding affinities were identified using IEDB. The top three peptides for the Y537S and D538G mutations, along with their associated wildtype peptides, were selected for in-vitro validation. T2 cells were pulsed with indicated peptide for four hours and surface HLA-A*0201 was stained. Median fluorescence intensity (MFI) of pulsed T2 cells were normalized to experimentally matched non-pulsed T2 cells, and data expressed as fold change in MFI for (A) Y537S peptides and (B) D538G peptides. Dashed horizontal line indicates non-pulsed T2 HLA-A*0201, also represented in grey as our control. Data represents 3 independent experiments performed in triplicate. Statistical significance was determined via comparison of pulsed MFI versus non-pulsed raw MFI using unpaired Student's t-test. *p<0.05, ***p<0.001.

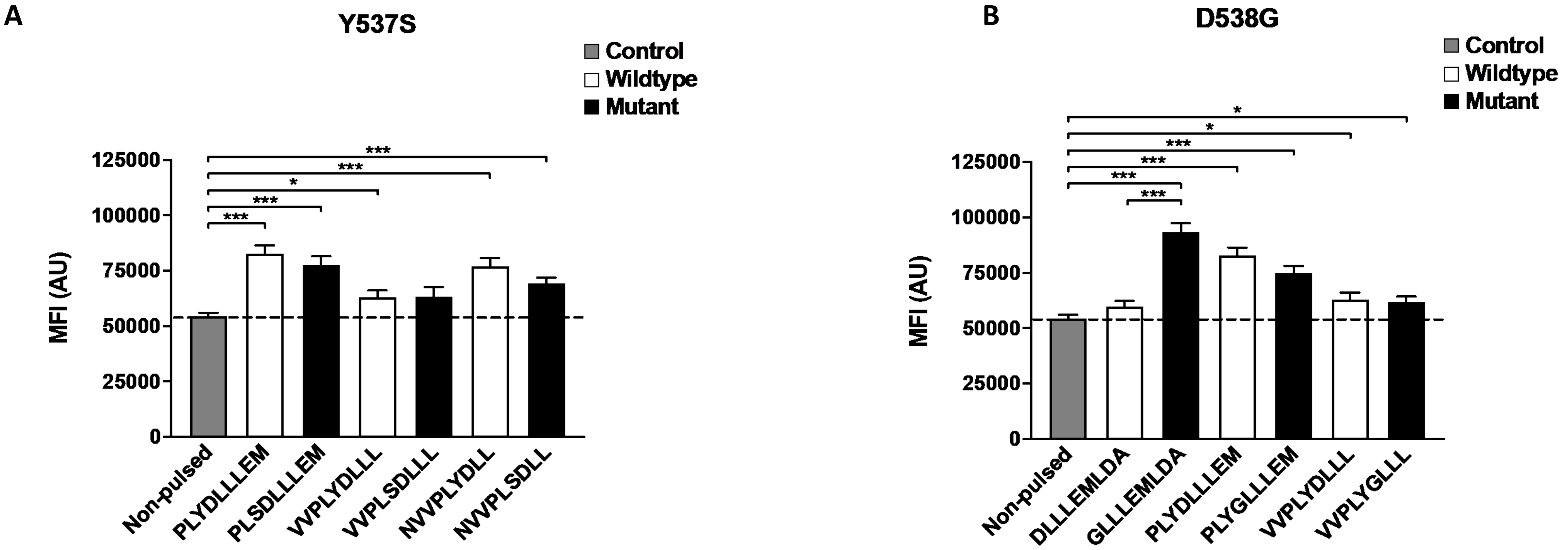

Supplement: Supplementary Figure S1 — Kinetics of Overlapping peptide [file crc-23-0244-s01.pdf]
