# Supplementary material for: Estrogen Receptor Mutations as Novel Targets for Immunotherapy in Metastatic Estrogen Receptor–positive Breast Cancer
Source: Cancer Res Commun. 2024 Feb 22;4(2):496–504. doi: 10.1158/2767-9764.CRC-23-0244 (PMC10883292; doi:10.1158/2767-9764.CRC-23-0244)
Supplement: Supplementary Table S1 — Initial in-silico identification of 18 novel peptides with predicted high affinity to HLA-A*0201 (IC50<500 nM) [file crc-23-0244-s02.pdf]

Supplementary Table S1

Supplementary Table S1: Initial in-silico identification of 18 novel peptides with predicted high affinity to HLA-A*0201 (IC50<500 nM).								
**Mutation**	**Mutant Epitope**	**IC50 (nM)**			**Wildtype Epitope**	**IC50 (nM)**		
		**NetMHCpan 4.0**	**ANN**	**SMM**		**NetMHCpan 4.0**	**ANN**	**SMM**
**E380Q**	LL**Q**CAWLEI	100.7	99.42	142.6	LLECAWLEI	379.1	394.08	432.62
**E380Q**	HLL**Q**CAWLEI	73.4	106.86	117.62	HLLECAWLEI	19.2	23.29	75.94
**V392I**	LMIGL**I**WRS	59.3	95.56	161.85	LMIGLVWRS	78.4	150.15	190.59
**V392I**	LMIGL**I**WRSM	104.4	23.42	759.42	LMIGLVWRSM	124.4	31.92	691
**S463P**	FL**P**STLKSL	78.3	45.94	104.5	FLSSTLKSL	31.6	25.04	70.49
**P535H**	**H**LYDLLLEM	13	14.73	46.68	PLYDLLLEM	170.3	353.62	255.33
**P535H**	**H**LYDLLLEML	19.5	31.88	46.08	PLYDLLLEML	270.7	526.76	232
**P535H**	V**H**LYDLLLEM	56.6	42.76	4260.69	VPLYDLLLEM	57.4	42.76	2867.35
**Y537S**	VPL**S**DLLLEM	163	135.01	3165.77	VPLYDLLLEM	57.4	42.76	2867.35
**D538G**	VPLY**G**LLLEM	120.4	97.36	2757.27	VPLYDLLLEM	57.4	42.76	2867.35
