# Supplementary material for: Estrogen Receptor Mutations as Novel Targets for Immunotherapy in Metastatic Estrogen Receptor–positive Breast Cancer
Source: Cancer Res Commun. 2024 Feb 22;4(2):496–504. doi: 10.1158/2767-9764.CRC-23-0244 (PMC10883292; doi:10.1158/2767-9764.CRC-23-0244)

Supplementary Figure S2 **Expression of ESR1 in various Healthy and Malignant Tissues.** mRNA expression data for multiple malignant and healthy tissues was obtained from TCGA and GTEx databases. Expression data are represented as TPM (transcripts per million). Healthy mammary tissue is labeled with a green arrow and invasive breast cancer samples are signified with a red arrow. Each sample is represented as a singular dot and each set statistically shown using inter quartile range box and whisper plots.

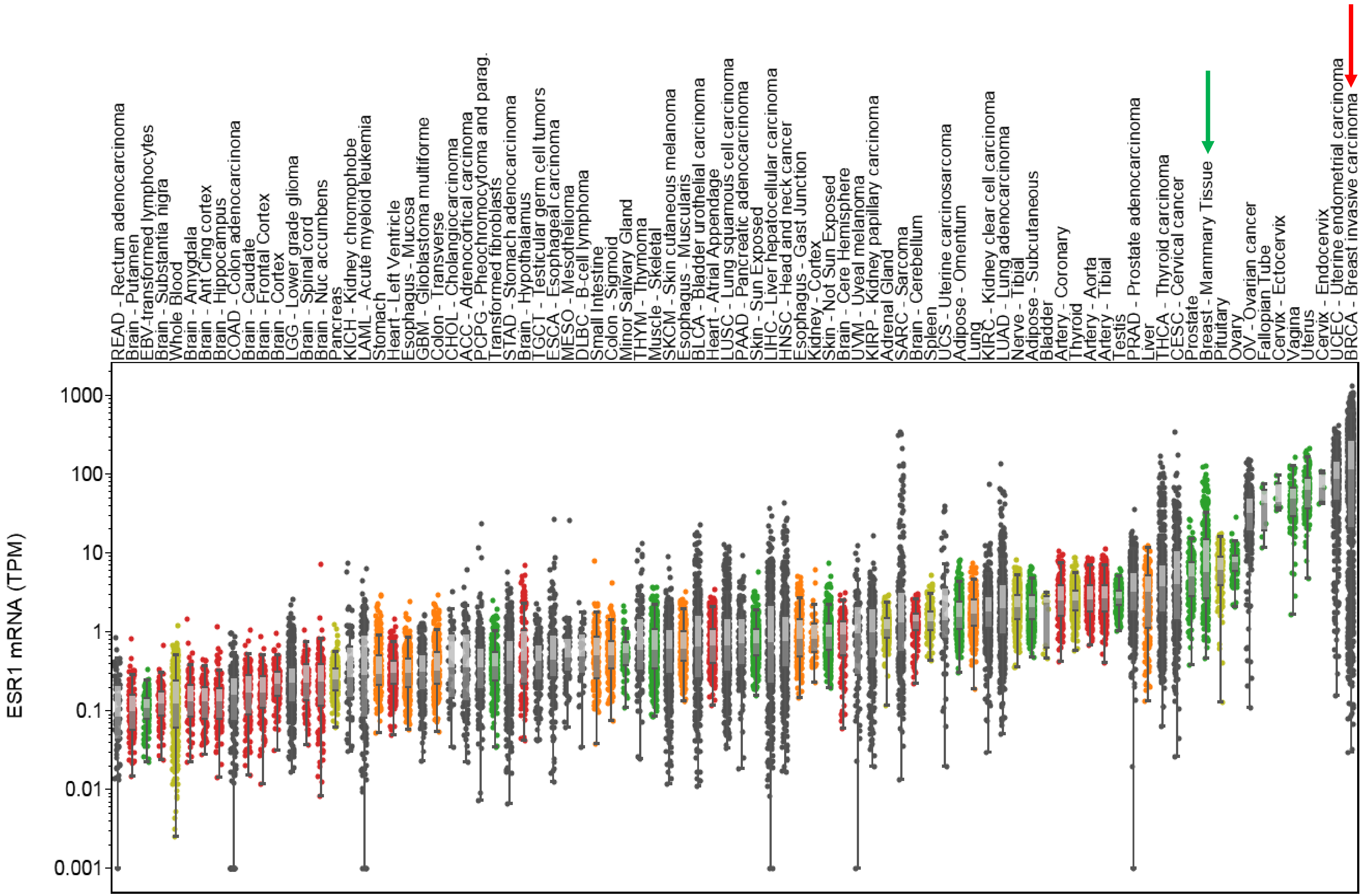

Supplement: Supplementary Figure S2 — Expression of ESR1 in various Healthy and Malignant Tissues [file crc-23-0244-s03.pdf]
