# Supplementary material for: Estrogen Receptor Mutations as Novel Targets for Immunotherapy in Metastatic Estrogen Receptor–positive Breast Cancer
Source: Cancer Res Commun. 2024 Feb 22;4(2):496–504. doi: 10.1158/2767-9764.CRC-23-0244 (PMC10883292; doi:10.1158/2767-9764.CRC-23-0244)
Supplement: Supplementary Table S2 — Clinically Validated Peptides with IC50>500nM [file crc-23-0244-s04.pdf]

Supplementary Table S2

Supplementary Table S2: Clinically Validated Peptides with IC50>500nM				
Peptide	Sequence	IC50 (nM)		
		NetMHCpan 4.1	ANN	SMM
MART-1	EAAGIGILTV	6687.84	5322.6	2220.65
gp100	KVPRNQDWL	15643.15	18993.7	5165.47
GP2	IISAVVGIL	1399.47	2514.94	1167.11
