# Supplementary material for: Estrogen Receptor Mutations as Novel Targets for Immunotherapy in Metastatic Estrogen Receptor–positive Breast Cancer
Source: Cancer Res Commun. 2024 Feb 22;4(2):496–504. doi: 10.1158/2767-9764.CRC-23-0244 (PMC10883292; doi:10.1158/2767-9764.CRC-23-0244)
Supplement: Supplementary Table S3 — Overlapping nonameric peptides in-silico prediction [file crc-23-0244-s05.pdf]

Supplementary Table S3

SupplementaryTable S3: Overlapping nonameric peptides in-silico prediction.								
Mutation	Overlapping Peptides	IC50 (nM)			Associated Wildtype	IC50 (nM)		
		NetMHCpan 4.0	ANN	SMM		NetMHCpan 4.0	ANN	SMM
**D538G**	**GLLLEMLDA**	**351.6**	**897.14**	**674.7**	**DLLLEMLDA**	**6970.9**	**8962.9**	**4467.97**
D538G	YGLLLEMLD	32442.2	23973.24	91224.2	YDLLLEMLD	38895.8	26458.9	91014.4
D538G	LYGLLLEML	22819.9	11161.6	12164.9	LYDLLLEML	15997.1	10414	5509.5
**D538G**	**PLYGLLLEM**	**347.2**	**1059.7**	**554.77**	**PLYDLLLEM**	**170.3**	**353.6**	**255.33**
D538G	VPLYGLLLE	35254.3	25030	426687.6	VPLYDLLLE	37111.9	26415.7	461434.4
**D538G**	**VVPLYGLLL**	**4576.3**	**4100.9**	**1599.96**	**VVPLYDLLL**	**3175.9**	**2057.3**	**1199.8**
D538G	NVVPLYGLL	6305.1	9063.5	5769.1	NVVPLYDLL	4165.1	5752.4	3381.5
D538G	KNVVPLYGL	15866.3	16628.5	12679.7	KNVVPLYDL	25980.6	20753.86	32070.8
D538G	CKNVVPLYG	42505.3	27804.9	395466.8	CKNVVPLYD	44210.8	31114.8	701632.99
Y537S	SDLLLEMLD	41766.3	31721.6	414104.5	YDLLLEMLD	38895.8	26458.9	91014.4
Y537S	LSDLLLEML	7764.2	6449	5213.3	LYDLLLEML	15997.1	10414	5509.5
**Y537S**	**PLSDLLLEM**	**907**	**1533.3**	**575.59**	**PLYDLLLEM**	**170**	**353.6**	**255.33**
Y537S	VPLSDLLLE	37528.6	28822.8	394557.22	VPLYDLLLE	37111.9	26415.7	461434.4
**Y537S**	**VVPLSDLLL**	**7885**	**5109.7**	**3742**	**VVPLYDLLL**	**3175.9**	**2057.3**	**1199.8**
**Y537S**	**NVVPLSDLL**	**4734.8**	**5931.9**	**2884.8**	**NVVPLYDLL**	**4165.1**	**5752.4**	**3381.5**
Y537S	KNVVPLSDL	31113.5	27874.2	133385.9	KNVVPLYDL	25980.6	20753.86	32070.8
Y537S	CKNVVPLSD	45657.1	33586.9	1462547.6	CKNVVPLYD	44210.8	31114.8	701632.99
Y537S	KCKNVVPLS	37359.3	29725.1	290475.8	KCKNVVPLY	36805.6	28794.7	692006.22
