# Supplementary material for: Estrogen Receptor Mutations as Novel Targets for Immunotherapy in Metastatic Estrogen Receptor–positive Breast Cancer
Source: Cancer Res Commun. 2024 Feb 22;4(2):496–504. doi: 10.1158/2767-9764.CRC-23-0244 (PMC10883292; doi:10.1158/2767-9764.CRC-23-0244)
Supplement: Supplementary Table S4 — Frequency of most common ESR1 mutations in key clinical studies [file crc-23-0244-s06.pdf]

Supplementary Table S4

Supplementary Table S4: Frequency of most common ESR1 mutations in key clinical studies.					
**Study**	**Total Missense Mutations**	**E380Q (%)**	**Y537S (%)**	**D538G (%)**	**Combined (%)**
FERGI	131	22 (17%)	26 (20%)	36 (27%)	84 (64%)
SOFEA	85	6 (7%)	16 (19%)	29 (34%)	51 (60%)
PALOMA-3	120	22 (18%)	23 (20%)	51 (42%)	96 (80%)
MSK-IMPACT	160	17 (11%)	34 (21%)	54 (34%)	105 (66%)
